# Large-scale production of foot-and-mouth disease virus (serotype Asia1) VLP vaccine in *Escherichia coli* and protection potency evaluation in cattle

**DOI:** 10.1186/s12896-016-0285-6

**Published:** 2016-07-02

**Authors:** Yan Xiao, Hong-Ying Chen, Yuzhou Wang, Bo Yin, Chaochao Lv, Xiaobing Mo, He Yan, Yajie Xuan, Yuxin Huang, Wenqiang Pang, Xiangdong Li, Y. Adam Yuan, Kegong Tian

**Affiliations:** National Research Center for Veterinary Medicine, Road Cuiwei, High-Tech District, Luoyang, 471003 People’s Republic of China; College of Animal Science and Veterinary Medicine, Henan Agricultural University, Zhengzhou, Henan 450002 People’s Republic of China; National University of Singapore (Suzhou) Research Institute, 377 Lin Quan Street, Suzhou Industrial Park, Jiangsu 215123 China; Department of Biological Sciences and Centre for Bioimaging Sciences, National University of Singapore, 14 Science Drive 4, Singapore, 117543 Singapore

**Keywords:** Foot-and-mouth disease, Serotype Asia 1, Virus-like particle vaccine, Fifty percent protection dose (PD_50_), Large scale production in *E. coli*

## Abstract

**Background:**

Foot-and-mouth disease (FMD) is an acute, highly contagious disease that infects cloven-hoofed animals. Vaccination is an effective means of preventing and controlling FMD. Compared to conventional inactivated FMDV vaccines, the format of FMDV virus-like particles (VLPs) as a non-replicating particulate vaccine candidate is a promising alternative.

**Results:**

In this study, we have developed a co-expression system in *E. coli*, which drove the expression of FMDV capsid proteins (VP0, VP1, and VP3) in tandem by a single plasmid. The co-expressed FMDV capsid proteins (VP0, VP1, and VP3) were produced in large scale by fermentation at 10 L scale and the chromatographic purified capsid proteins were auto-assembled as VLPs in vitro. Cattle vaccinated with a single dose of the subunit vaccine, comprising in vitro assembled FMDV VLP and adjuvant, developed FMDV-specific antibody response (ELISA antibodies and neutralizing antibodies) with the persistent period of 6 months. Moreover, cattle vaccinated with the subunit vaccine showed the high protection potency with the 50 % bovine protective dose (PD_50_) reaching 11.75 PD_50_ per dose.

**Conclusions:**

Our data strongly suggest that in vitro assembled recombinant FMDV VLPs produced from *E. coli* could function as a potent FMDV vaccine candidate against FMDV Asia1 infection. Furthermore, the robust protein expression and purification approaches described here could lead to the development of industrial level large-scale production of *E. coli*-based VLPs against FMDV infections with different serotypes.

## Highlights

1. A co-expression system in *E. coli* driving the expression of FMDV capsid proteins (VP0, VP1, and VP3) in tandem by a single plasmid is constructed.
2. Large-scale chromatographic purified FMDV capsid proteins are auto-assembled into VLPs in vitro.
3. VLP-based FMDV vaccine induces strong and persistent humoral immunity with the period of 6 months
4. VLP-based FMDV vaccine displays strong protective efficacy against FMDV challenge

## Background

Foot-and-mouth disease (FMD) is a highly contagious and devastating disease of cloven-hoofed animals that causes significant economic losses worldwide [1]. FMD is endemic in many countries including parts of Asia, Africa, South America, and at the periphery of the European Union [2]. The causative agent, foot-and-mouth disease virus (FMDV), belongs to the genus *Aphthovirus* of the family *Picornaviridae*. The virus consists of seven serotypes, including type A, O, C, Asia 1, and South African Territories (SAT) 1–3 [3].

Conventional inactivated whole-virus vaccines are the primary measure for controlling the disease in most endemic areas [3]. Such vaccines have played important roles in eliminating the disease from some areas of the world [4]. However, potential dangers still accompany the use of whole virus vaccines because of the incomplete inactivation of FMDV in large-scale production and the risk of escape of the live virus from vaccine producing facilities [5]. Thus, a number of different types of vaccines have been developed including synthesized peptide vaccines [6], recombinant virus-vectored vaccines [7], virus-like particles (VLPs) subunit vaccines [8–10], and DNA vaccines [11, 12]. Among these, the VLP subunit vaccine that contains all of the immunogenic sites present on the intact virions but lacks nucleic acid component is safe and effective. Development of VLP vaccines has been attempted by using various expression platforms including eukaryote and prokaryote expression systems [8, 13]. Compared to the eukaryote expression system, the production of recombinant proteins in the prokaryote system, such as *E. coli*, is considered to be less laborious and less expensive.

The FMDV genome contains a single large open reading frame. The capsid of FMDV consists of three structural proteins VP0, VP1 and VP3, which are the cleavage products of the P1-2A capsid precursor polypeptide yielded from viral 3C protease digestion. During virus maturation, capsid protein VP0 is further cleaved to make capsid proteins VP2 and VP4. Either capsid proteins VP0, VP1 and VP3 or capsid proteins VP1, VP2, VP3 and VP4 are able to form the icosahedral capsid particles during the different stages of virus maturation by self-assembly [8]. In addition, two research groups successfully produced empty icosahedral capsid particles by expression of VP0, VP1 and VP3 capsid proteins in *E. coli* by fusing these proteins with small ubiquitin-like modifier (SUMO) tag [8, 14]. Furthermore, Guo et al. [8] evaluated the immunogenicity of the in vitro assembled VLPs in cattle, which is composed of capsid proteins VP0, VP1 and VP3. However, both published methods used 2 and 3 plasmids with different antibiotic selection markers, respectively, to successfully co-express the FMDV capsid protein in soluble form. However, the multiple antibiotic selection pressure by using multiple vectors with different antibiotic selection markers could significantly reduce the bacterial growth and could raise environmental and food security concerns. Moreover, the VLP preparation protocols provided by these two papers may not be suitable for large scale production of VLP vaccine at industrial level since neither analytical size exclusion chromatographic approach nor sucrose gradient ultracentrifugation approach was considered as industrially favorable one for vaccine preparation. Furthermore, the reported yield of VP0-VP1-VP3 ternary complexes was ~5 mg/L by using two plasmids and even less yield of ternary complexes was achieved by using three plasmids [8, 14].

In this study, we optimized the *E. coli* co-expression system by co-expressing SUMO fused full-length FMDV capsid proteins (VP0, VP1, and VP3) in tandem driven by a single plasmid and selected by a single antibiotic. In our case, the co-expressed FMDV capsid proteins (VP0, VP1, and VP3) were produced by fermentation at 10 L scale with the protein level reaching ~50 mg/L culture without further optimization. Furthermore, we optimized the purification protocols by obtaining ~90 % pure FMDV capsid proteins without size exclusion chromatographic purification, which is not preferred at industrial level due to the cost. The expressed full-length capsid proteins VP0, VP1, and VP3 were in vitro assembled into VLPs, which showed high protection potency with 11.75 PD_50_ per dose when applied as subunit vaccines in cattle. Taken together, our data provided a robust protocol, for the first time, leading to large-scale production of potent FMDV VLP vaccines against FMDV Asia1 infection.

## Methods

### Production of recombinant protein and characterization of VLPs

The full-length FMDV VP0, VP1 and VP3 genes were synthesized (Genewiz) and cloned into the plasmid pETSUMO, designated as pETSUMO-VP0, pETSUMO-VP1, and pETSUMO-VP3, respectively. Subsequently, the respective DNA fragments of the clones including the ribosome binding site, the SUMO and the FMDV VP gene, designated as RBS-SUMO-VP3, RBS-SUMO-VP1, and RBS-SUMO-VP0, were amplified by PCR from pETSUMO-VP3, pETSUMO-VP1 and pETSUMO-VP0, respectively. Then these DNA fragments were cloned into a single pET28b vector (Novagen, USA) in order. The recombinant plasmid obtained was designated as pET-TRI-Asia1-VP310. All the restriction enzymes were purchased from New England Labs and the polymerases were purchased from Qiagen.

The recombinant plasmid pET-TRI-Asia1-VP310 was transformed into *E. coli* BL21 (DE3) competent cells (Stratagene, USA) according to the manufacturer’s manual. A single colony of transformant was grown in Luria-Bertani (LB) medium containing 50 μg/ml kanamycin at 37 °C until the OD_600_ reached 0.8. Then isopropyl β-D-thiogalactopyranoside (IPTG) was added to a final concentration of 0.4 mM. The culture was incubated for 4 h at 28 °C before subjected to sodium dodecyl sulfate polyacrylamide gel electrophoresis (SDS-PAGE) and western-blot to confirm the expression of recombinant protein.

Next, the fermentation was performed in 10 L bioreactor (Baoxing, Shanghai, China). The media for the primary seed, secondary seed, and fermentation of the cultures were LB comprising tryptone 10 g/L, yeast extract 5.0 g/L, NaCl 10 g/L, and kanamycin 50 g/L. The primary seed culture was prepared by the transfering of 1 mL of glycerol stock of recombinant *E. coli* strain to 50 mL of LB medium in a 250-mL flask, which was then aerobically incubated overnight at 37 °C. The secondary seed culture was prepared by inoculating 2 L flasks containing 500 mL of LB medium with 5 mL of primary seed culture and cultivating overnight at 37 °C and 220 rpm. Flask of the secondary seed culture were then inoculated into 5.0 L of medium in a 10-L bioreactor and cultivating at 37 °C and pH 7.0, in which dissolved oxygen was maintained above 20 %. The fed-batch process of recombinant protein production in 10-L bioreactor was divided into three phases. Phase I was an aerobic batch cultivation at 37 °C for 2 h. Phase II was a fed-batch cultivation at 37 °C for 4 h in which 300 g/L concentrated glucose was fed continuously to maintain the required specific growth rate of bacteria. In Phase III was an induction process at 37 °C for 6 h using isopropyl-β-d-thiogalactoside (IPTG) at a final concentration of 0.4 mM to induce the expression of recombinant protein. The bacteria were harvested by centrifugation and the cell pellets were re-suspended at the buffer A (300 mM NaCl, 20 mM Tris–HCl [pH7.0]). The cells were disrupted by homogenizer (Avestin) and the cell supernatant was run through the His-affinity chromatographic purification, washed by buffer B (50 mM Tris–HCl, 0.3 M NaCl, 0.2 mM EGTA, and 40 mM imidazole) and eluted by using 500 mM imidazole. The SUMO protein tag was removed by Ulp1 digestion, followed by Q Sepharose Fast Flow anion exchange chromatographic purification (GE Healthcare). The purified proteins were concentrated by using Amicon centrifuge tubes (Millipore) for small scale preparation or by using Centramate TFF cassette (PALL) for large scale production. The purified recombinant FMDV VP3, VP1 and VP0 proteins were resuspended with 50 mM Tris–HCl, supplemented by a series of NaCl with different concentrations. The purified recombinant FMDV VP3, VP1 and VP0 proteins have been almost completely re-assembled into VLPs in a buffer of 50 mM Tris–HCl and 500 mM NaCl.

### Transmission electron microscope

Purified VLPs were adsorbed onto a copper grid for 5 min at room temperature. The grids were dried gently using filter paper and stained with 3 % of phosphotungstic acid (PTA) for 5 min. The excess liquid was removed with filter, and the samples were examined under a TEM at 80 kV (FEI).

### Dynamic Light Scattering (DLS) analysis of FMDV capsid VLPs

Dynamic light scattering studies were carried out on a DynaPro Light Scattering instrument (Wyatt Technology Europe GmbH, Dernbach, Germany) with protein concentrations at A280 nm of 1.0, in buffer containing 50 mm sodium phosphate, pH 6.5, and 5 mm DTT.

### Vaccine preparation

The purified capsid protein and adjuvant ISA 206 (SEPPIC, France) was emulsified using a RW 20 homogenizer (IKA, German) under sterile conditions at 300 rpm for 5 min, in a ratio of 46:54 (antigen:adjuvant) in volume. The preparations were stored at 4 °C until use.

### Animals and experimental design

#### Vaccination and antibody monitor in cattle

Ten six-month-old cattle, sero-negative of FMDV (LPBE antibody titers <1: 16) were randomly assigned to two groups with 5 animals in each group. Group A and group B were vaccinated intramuscularly once with 2 ml of FMDV Asia1 subunit vaccine containing 200 μg and 100 μg VLPs per cattle, respectively. Serum samples were collected at 21, 60, 90, 120, 150 and 180 dpv, respectively.

#### Fifty percent protection dose (PD_50_) test

Seventeen six-month-old cattle, sero-negative of FMDV (LBPE antibody titers ≤1: 8), were divided into four groups, and all animals were housed in an animal biosafety level 3 (BSL3) facility. Groups 1, 2 and 3 with 5 animals in each group were vaccinated intramuscularly one dose subunit vaccine, 1/3 dose subunit vaccine, and 1/9 dose subunit vaccine, respectively; Group 4 with 2 animals, sham-vaccinated with PBS, was used as challenge control.

PD_50_ test was performed as described by the OIE to test the potency of FMDV VLPs produced from *E. coli* as a vaccine candidate. All cattle were challenged by tongue intradermal inoculation with 10,000 bovine infective dose 50 % (ID_50_) of FMDV strain Asial/JSL/GSZY/06 per head at 28 days post vaccination (dpv). The animals were daily examined for possible occurrence of lesions in the mouth and feet. Any lesion at a site other than the inoculation site within 10 days post-challenge was considered as a rupture of immunity and considered as no protection.

Serum samples were collected at 0, 7, 14, 21, and 28 dpv. The bovine PD_50_ content of the vaccine was calculated based on the Reed-Muench method from each animal protected in each group. The animal trial in this study was approved by the Animal Care and Ethics Committee of China National Research Center for Veterinary Medicine with reference number 2015062503, and conventional animal welfare regulations and standards were taken into account.

### Serology test

The FMDV-specific antibody titers in immunized cattle were determined by a commercial liquid-phase-block ELISA (LPB-ELISA) kit (Lanzhou Veterinary Research Institute, China). Virus neutralization test (VNT) was performed according to the protocol described by the OIE. Serum samples were inactivated at 56 °C for 30 min and subjected to a microtiter neutralization assay on BHK-21 cells. Two-fold serially diluted sera were incubated with 100 TCID_50_ of FMDV strain Asial/JSL/GSZY/06 at 37 °C and 5 % CO_2_ for 1 h, followed by infection of monolayers of BHK-21 cells in 96-well plates for 72 h. Thereafter, the cells were examined for FMDV-specific cytopathic effect (CPE), and neutralization titers were calculated based on the Reed-Muench method.

### Statistical analysis

All data were expressed as the mean value of five animals ± SEM. Differences were considered statistically significant when *P* < 0.05.

## Results

### Expression, purification, and characterization of FMDV VLPs

**Fig. 1:**
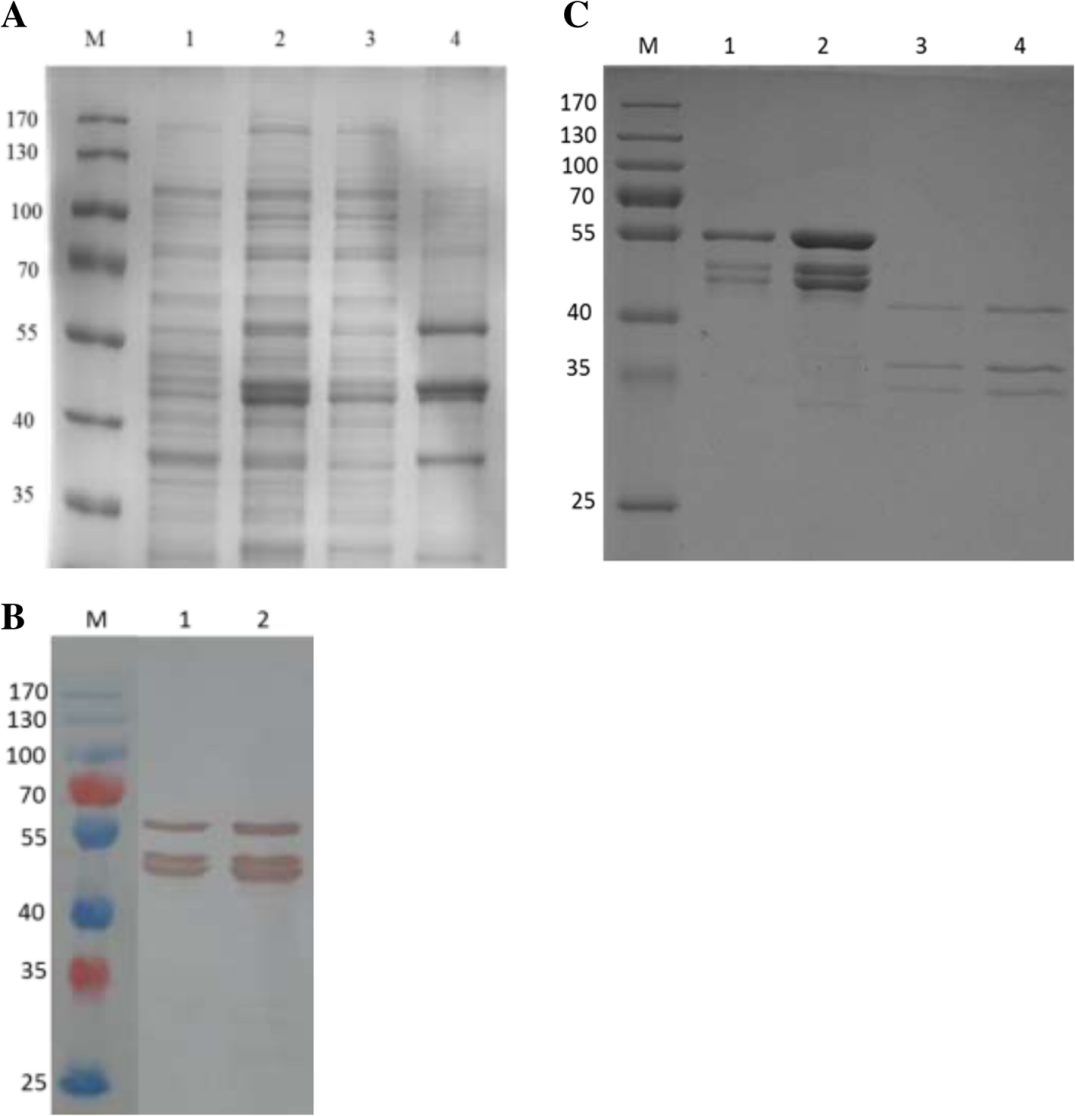
SDS-PAGE and western-blot analysis of FMDV capsid proteins expressed in *E. coli.* **a** SDS-PAGE of FMDV capsid protein expression without purification. M, protein marker; lane 1, total protein without IPTG induction; lane 2, total protein after IPTG induction; lane 3, supernatant protein after IPTG induction; lane 4, pellet protein after IPTG induction; **b** Western-blot of FMDV capsid protein expression without purification. M, protein marker; lane 1, supernatant protein after IPTG induction; lane 2, pellet protein after IPTG induction; **c** M, protein marker; lane 1, purified protein complex (1ul load); lane 2, purified protein complex (10ul load); lane 3, purified protein complexes after Ulp 1 protease treatment (1ul load); lane 4, purified protein complexes after Ulp 1 protease treatment (10ul load)

**Fig. 2:**
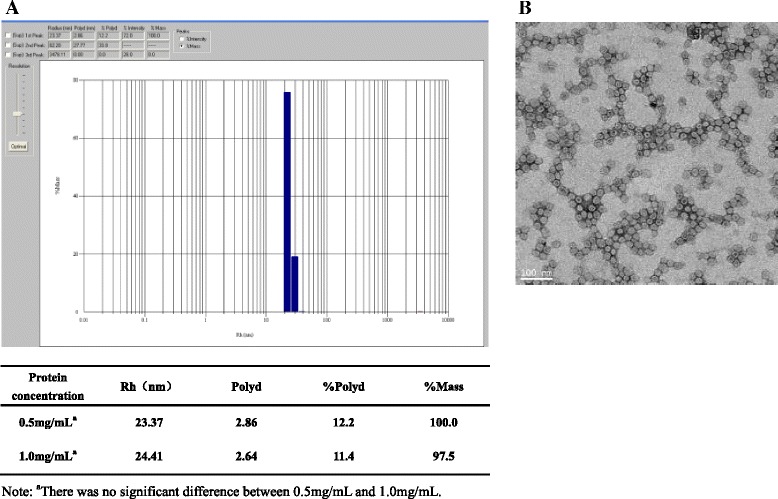
Characterization of in vitro re-assembled FMDV VLPs. **a** DLS profile. The difference of DSL profile between two protein concentrations was determined by using *t*-test in GraphPad 5.0. Differences were considered statistically significant when P < 0.05. **b** Images of VLPs under electron microscope with 42000 × magnification. The scale bar indicates 100 nm

VP0, VP1 and VP3,the capsid proteins of FMDV, have the ability to form empty capsids resembling the capsids of virions and share the same antigenicity as virions. To obtain the FMDV VLPs, the recombinant plasmid pET-TRI-Asia1-VP310 containing the full-length of VP0, VP1 and VP3 genes in tandem was constructed. The recombinant expression vector was then transformed into BL21/DE3 competent cells and protein expression was induced by IPTG. SDS-PAGE results showed that the recombinant proteins of expected size were expressed (Fig. 1). As shown in Fig. 1a, the recombinant proteins were found in the supernatant of cells after disruption by homogenizer (Avestin), which is about 20 % of the total soluble proteins. Western-blot results showed that the recombinant proteins reacted with FMDV positive serum (Fig. 1b). The soluble fractions from bioreactor massive production were purified by affinity chromatography with Ni^2+^ resins (Novagen, USA) to capture the His × 6-fusion proteins. After washing with washing buffer (50 mM Tris–HCl, 0.3 M NaCl, 0.2 mM EGTA, and 40 mM imidazole), the target proteins were eluted by 500 mM imidazole and treated with SUMO protease Ulp1 overnight at 4 °C to remove the SUMO tag. The digestion products were further purified by Q Sepharose Fast Flow anion exchange chromatographic purification. The total purification yield is around 20 ~ 35 % (Table 1). SDS-PAGE showed that the purity of the final recombinant capsid protein reached ~90 % calculated by Quantity One (Bio-Rad) (Fig. 1c). The purified recombinant capsid proteins (VP0, VP1 and VP3) were assembled into VLPs in vitro. Dynamic light Scattering (DLS) results showed that almost 100 % of purified capsid proteins form uniform high molecular weight complex, suggesting VLP formation (Fig. 2a). The assembled VLPs were diluted in 50 mM Tris–HCl and submitted to TEM analysis, which showed that the in vitro assembled FMDV VLPs were rendered as hollow shapes with the diameters of ~30 nm with 42 000 fold magnification which consistent with the DLS results (Fig. 2b).

**Table 1:** Protein yield at different purification steps

Steps	Procedure	Protein recovery (%)
1	Ni2+ affinity chromatography to capture SUMO-VP proteins	70 %-80 %
2	Cleavage of SUMO tag using Ulp1 to release the un-tagged VP proteins	60 %-70 %
3	Q Sepharose Fast Flow anion exchange chromatography to obtain the pure VP proteins	50 %-60 %
	Total yield:	20 %-35 %

### Immune-reactivity in cattle induced by vaccination with VLP vaccine

**Fig. 3:**
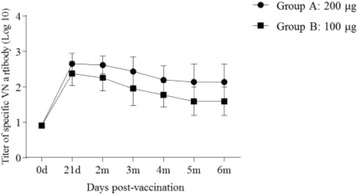
FMDV-specific immune responses in cattle measured by LPB-ELISA. Groups of cattle (n = 5) immunized with 200ug or 100ug of FMDV VLPs. Blood samples were collected at 21 days post-vaccination and 2, 3,4,5 and 6 months after vaccination for the LPB-ELISA detection. LPB-ELISA value ≥ 128 (log_10_ 2.1) indicates above 99 % protection to the viral challenge according to the manufacture’s instruction

Cattles were divided into two groups (groups A and B) and submitted to vaccination with 200 μg and 100 μg FMDV VLP per cattle, respectively. The specific anti-FMDV antibody responses were evaluated by commercial LPB-ELISA kits. As shown in Fig. 3, the specific antibodies were detected at 21 dpv in all vaccinated animals, and the antibody levels decreased gradually thereafter (Fig. 3). The persistent periods (mean titer ≥128) of FMDV-specific LPB antibodies in groups A and B were 6 and 3 months, respectively.

**Fig. 4:**
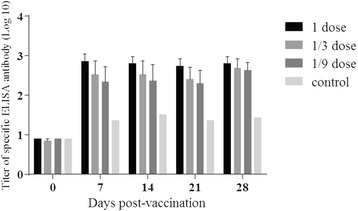
FMDV-specific immune responses in cattle measured by LPB-ELISA in PD_50_ test. Blood samples were collected at 0, 7, 14, 21, and 28 days post-vaccination for the LPB-ELISA detection. LPB-ELISA value ≥ 128 (log_10_ 2.1) indicates above 99 % protection to the viral challenge according to the manufacture’s instruction. Each data points represents the mean ± standard error of measurements for five cattle from immunized groups and for 2 cattle from control group

**Fig. 5:**
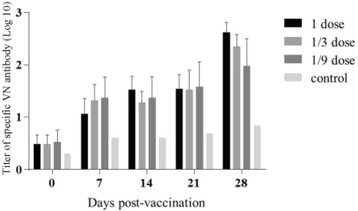
FMDV-specific immune responses in cattle measured by virus neutralizing antibody test. Blood samples were collected at 0, 7, 14, 21, and 28 days post-vaccination for the virus neutralizing antibody test. The virus neutralizing antibody was expressed by the average titers of cattle in one whole group at different time points. Each data points represents the mean ± standard error of measurements for five cattle from immunized groups and for 2 cattle from control group

To further investigate the correlations between the dose and the persistent period of FMDV-specific antibodies, three more groups of cattle were inoculated with one dose (200 μg), 1/3 dose, and 1/9 dose of FMDV VLP vaccine, respectively. FMDV-special ELISA and neutralization antibody titers were determined at 0, 7, 14, 21, and 28 dpv for each cattle. As shown in Fig. 4, all 5 animals vaccinated with 1 dose and most of the 10 cattle vaccinated with 1/3 dose and 1/9 dose FMDV VLP vaccine developed a detectable level FMDV ELISA antibody response at 7 dpv with the titer reaching up of 1440 (Fig. 4). Notably, the antibody titers were always maintained at high levels for the whole experimental period from 7dpv till 28 dpv. Consistently, FMDV-specific neutralizing antibodies were detected only in few serum samples at 7 dpv, which increased gradually to reach the highest level at challenge (28 dpv) (Fig. 5). In contrast, the antibody titers in the control group were negative for the both LPBE and VNT groups.

### PD_50_ test using Asia1/JSL/GSZY/06

The PD_50_ test was performed to assess the subunit vaccine potency by following the bovine potency test protocol described by the OIE to test the traditional inactivated FMDV vaccines. When challenged with FMDV strain Asial/JSL/GSZY/06, both the control animals exhibited typical FMD lesions on at least three feet by 5 day post-challenge (dpc), while vaccinated animals showed different levels of protection. All five cattle were considered completely protected in the 1 dose immunized group, three of the four cattle were protected in the 1/3 dose group as one cattle in this group died after challenge with no typical lesions of FMD, and four of the five cattle were protected in the 1/9 dose group. The lesions in the 1/3 dose and 1/9 dose groups were detectable by 6 dpc and the lesions were less severe compared to control group with lesion on only one foot. In this research, the result showed the vaccine potency of the batch immunized with the expressed antigens reached 11.75 PD_50_ per dose (Table 2). These data indicated that the VLPs produced in *E. coli* elicited a protective immune response in cattle.

**Table 2:** Results of PD_50_ test. PD_50_ test was performed as described by the OIE. The bovine PD_50_ content of the vaccine was calculated based on the Reed-Muench method from each animal protected in each group

Groups	Animal No.	Antibody titer at 28 dpv		Protection	Days of onset of lesion	Rate of protection	PD_50_
		LPBE	VN	Yes	-	5/5	
		LPBE	VN	Yes	-	5/5	11.75
1 dose	1558	360	64	Yes	-		
	1559	1440	64	Yes	-		
	1584	1440	724	Yes	-		
	1601	180	362	Yes	-		
	1602	720	512	Yes	-		
1/3 dose	1534	1440	724	Yes	-	3/4	
	1548	1440	724	No	D10		
	1562	90	724	Death^a^	-		
	1567	720	431	Yes	-		
	1576	180	76	Yes	-		
1/9 dose	29	1440	724	Yes	-	4/5	
	77	360	54	Yes	-		
	1136	2880	724	Yes	-		
	1139	180	4	No	D6		
	1580	360	512	Yes	-		
Control	1561	22	16	No	D5	0/2	
	1590	45	8	No	D5		

^a^ One animal in this group died after challenge, but with no typical lesions of FMD

## Discussion

FMD, one of the most devastating diseases of livestock, can cause significant economic losses worldwide, and remains the most important constraint to international trade in live animals and animal products. FMDV is divided into seven serotypes with no cross-protection conferred among the serotypes, and three of which exist in China, including serotypes O, Asia1, and A. FMDV serotype Asia1, an unique serotype of Asia, was first detected in samples collected in India in 1951 through 1952 and Pakistan in 1954 [15]. In China, serotype Asia1 became an important prevalent serotype after an outbreak recorded in Hong Kong in 2005 [16]. FMD vaccines are applied for prophylactic use in endemic areas worldwide, two vaccine types including traditional killed vaccines and synthetic peptide vaccines are commercially available and widely used in China.

Although the traditional inactivated vaccine has been proved effective, the development of a novel subunit FMDV vaccine that is safer, more effective and more economical than traditional vaccines is essential to control FMDV spreading worldwide. Notably, FMDV is a non-enveloped RNA virus and each FMDV capsid subunit is formed by the assembly of a single copy of the structural proteins VP0, VP1, and VP3 coded by the P1 region of the viral genome. Among them, VP0 is digested to make capsid proteins VP2 and VP4 during virus maturation [17]. In previous reports, the fusion proteins of three FMDV capsid proteins, VP0, VP1 and VP3, have been successfully expressed in *E. coli* as SUMO-fusion format at ~5 mg/L level by co-transformation of 2 or 3 plasmids with multiple antibiotic selections [8, 14]. No robust protocols for large scale production and purification of FMDV VLPs were provided. In this study, we first made a single plasmid by constructing RBS-SUMO-VP3, RBS-SUMO-VP1 and RBS-SUMO-VP0 genes in tandem and driven by a single promoter with its own ribosome binding site, which led to the co-expression of SUMO-tagged VP3, VP1 and VP0 in *E. coli*, selected by only one single antibiotics. Compared with the previous reported production of FMDV vaccines, our protocol has the following advantages. 1) The genes encoding SUMO-tagged VP3, VP1 and VP0 were constructed in tandem into a single plasmid and selected by a single antibiotics; 2) The SUMO-tagged VP3, VP1 and VP0 proteins in this protocol were expressed at soluble form with the expression level as high as ~50 mg/L by fermentation, which was ten times higher than the previously reported protocols [8, 14]; 3) The addition of SUMO tags not only facilitates the ensuing purification processes but preventing the aggregation of FMDV capsid proteins; 4) A robust purification protocol was provided to make well-assembled FMDV VLPs with the purify reaching ~90 % without using the industrial non-preferred size exclusion chromatographic purification or sucrose gradient ultracentrifugation steps, which dramatically reduced the cost of vaccine production. Moreover, the application of our in vitro assembled FMDV VLP vaccine has successfully elicited robust high level of VN antibodies in the tested cattle right after 28 dpv. Furthermore, high level of VN antibodies were detected in all cattle of 1 dose group and 1/3 dose group, and four out of five in 1/9 dose group (Table 1). Unexpected, animal No. 1548 in 1/3 dose group displaying a high VNA titer showed no resistance to challenge. However, similar observation was also seen in previous studies of FMDV VLP subunit vaccine development [6, 8]. Although the good correlation between VNA titer and protection to FMDV challenge was generally considered, the disparity sometimes was reported due to variation of individual cattle in resistance to the virus infection as well as other immunological parameters such as cellular immunity.

Surprisingly, in the PD_50_ test, the percentage protection values showed a dose independent manner since either 1 dose, 1/3 dose or 1/9 dose FMDV VLP subunit vaccine displays similar significant protection. This observation is also consistent with the earlier reports in the FMDV potency tests [18, 19]. As reported by Goris et al. [18], the largest variation in the number of protected animals was observed in the lowest vaccine volume dose group, where the entire possible range of protected animals varied widely (from 0 to 5). Nevertheless, our *E. coli*-based FMDV VLP vaccines produced by large-scale fermentation have shown the great promise in protecting cattle from FMDV infection.

Notably, according to the World Organization for Animal Health (OIE) manual (OIE, 2012), the FMDV vaccine should contain at least 3 PD_50_ and 6 PD_50_ per dose for cattle when employed for routine prophylactic vaccination and emergency vaccination, respectively. In China, Ministry of Agriculture of China elevated the potency criteria of all commercial FMDV vaccines from 3 to 6 PD_50_ per dose in 2013 (http://www.moa.gov.cn/zwllm/tzgg/tfw/201306/t20130613_3491208.htm). Such standard demands the development and production of FMDV vaccines with potent protection. Strikingly, our *E. coli*-based FMDV VLP vaccines by fermentation achieve 11.75 PD_50_ per dose in cattle. Hence, our approach in large scale production of FMDV VLP subunit vaccine by *E. coli* expression system should have direct impact in controlling FMD spreading worldwide.

## Conclusions

In this study, the FMDV capsid proteins VP0, VP1, and VP3 were expressed in *E. coli* and assembled into VLPs in vitro in large scale by fermentation at 10 L scale. VLP-based FMDV vaccine induced strong and persistent humoral immunity with the period of 6 months which indicated this vaccine candidate could function well against FMDV Asia1 infection.

## Abbreviations

DLS, dynamic light scattering; *E.coli, Escherichia coli*; FMDV, foot-and-mouth disease virus; IPTG, isopropyl-β-d-thiogalactoside; PD_50_, 50 % bovine protective dose; SDS-PAGE, sodium dodecyl sulfate polyacrylamide gel electrophoresis; VLP, virus-like particle
